# Professor Gibson

**Published:** 1854-11

**Authors:** 


### PROFESSOR GIBSON.

It has been stated in several Medical Journals that Prof. Gibson had resigned the chair of Surgery in the University of Pennsylvania. We learn that this announcement is premature. No change has taken place in the Faculty of that School since the close of the last session.

